# Unraveling the challenges of intravenous leiomyomatosis: a retrospective analysis of 11 cases

**DOI:** 10.1007/s00404-023-07308-x

**Published:** 2023-12-12

**Authors:** Qun Wang, Hua Liu, Weiwei Feng

**Affiliations:** Department of Gynecology and Obstetrics, Ruijin Hospital, Shanghai Jiao Tong University School of Medicine, 197 Ruijin Er Road, Shanghai, 200025 China

**Keywords:** Intravenous leiomyomatosis, IVL, Leiomyoma, Uterine fibroids

## Abstract

**Objective:**

This study provides a concise overview of diagnostic and treatment strategies for intravenous leiomyomatosis (IVL), a rare disease with nonspecific clinical manifestations, based on cases from a tertiary referral hospital in China.

**Methods:**

We retrospectively analyzed 11 premenopausal patients with confirmed IVL between 2018 and 2022. Clinical data from Ultrasound, Enhanced CT, and MRI were studied, along with surgical details, postoperative pathology, and follow-up information.

**Results:**

Premenopausal patients showed no disease-specific symptoms, with 90.9% having a history of gynecological or obstetric surgery, and 72.7% having prior uterine fibroids. Cardiac involvement was evident in two cases, with echocardiography detecting abnormal floating masses from the inferior vena cava. Pelvic ultrasound indicated leiomyoma in 90.9% of cases, with ≥ 50 mm size. Surgery was the primary treatment, and lesions above the internal iliac vein resulted in significantly higher intraoperative blood loss (median 1300 ml vs. 50 ml, *p* = 0.005) and longer hospital stays (median 10 days vs. 4 days, *p* = 0.026). Three patients with lesions above the inferior vena cava required combined surgery with cardiac specialists. Recurrence occurred in 2 out of 11 patients with incomplete lesion resection.

**Conclusions:**

IVL mainly affects premenopausal women with uterine masses, primarily in the pelvic cavity (Stage I). Pelvic ultrasound aids early screening, while Enhanced CT or MR assists in diagnosing and assessing venous lesions. Complete resection is crucial to prevent recurrence. Lesions invading the internal iliac vein and above pose higher risks during surgery. A multidisciplinary team approach is essential for patients with lesions above the inferior vena cava, with simultaneous surgery as a potential treatment option.

## What does this study add to the clinical work

**Table:** 

This study adds valuable insights to the clinical work by highlighting the nonspecific clinical manifestations of intravenous leiomyomatosis (IVL) in premenopausal women with a history of gynecological or obstetric surgery and prior uterine fibroids, emphasizing the importance of using imaging modalities for accurate diagnosis, and underscoring the significance of a multidisciplinary team approach for managing complex cases involving critical vascular structures.	

## Introduction

Intravenous leiomyomatosis (IVL) is a rare benign disease but a biologically aggressive tumor [1]. It usually originates in the uterus and extends into the extrauterine venous system and can even reach the right atrium/ventricle or pulmonary artery [2, 3]. According to the disease progression, IVL is divided into four stages [4]: Stage I: the lesion was confined to the pelvic cavity; Stage II: lesions located in the iliac vein/inferior vena cava; Stage III: the tumor entered the right atrium/ventricle; Stage IV: The tumor reaches the pulmonary artery [4]. IVL usually has no specific symptoms, when it grows into the heart cavity, symptoms such as palpitation, chest tightness, and syncope may occur [1, 2, 5], even sudden death as a result of total cardiac obstruction [3, 6], which is often confused with cardiovascular diseases. The best treatment of IVL is complete tumor resection combined with a hysterectomy and Bilateral salpingo-ophorectomy [4, 7–9]. The removal of circulatory tumors has always been a complicated operation, and multi-disciplinary treatment is usually required. For clinicians who are unfamiliar with the above condition, it is easy to misdiagnose, here, we summarized the diagnosis and treatment experience of 11 patients with IVL in our center during the past 5 years.

## Patients and methods

A total of 11 patients were included in this study, all of whom were diagnosed with IVL through histopathology at Ruijin Hospital Affiliated with Shanghai Jiao Tong University School of Medicine, between 2018 and 2022. All patients underwent surgical treatment at the Department of Gynecology and Obstetrics. Except for 2 patients (Case 8, Case 9) who initially sought care from a cardiologist due to symptoms of chest tightness, all others first consulted a gynecologist. Comprehensive medical records of each patient were reviewed, and all patients were followed up.

The review encompassed past medical history, surgical data, patient demographics, clinical symptoms, imaging findings, surgical procedures, postoperative conditions, pathological data after surgery, and follow-up outcomes.

### Statistical analysis

Statistical analysis was conducted using the Statistical Package for Social Science (SPSS) version 22.0 for Windows (SPSS, Inc., Chicago, Illinois). Nonparametric methods were employed to analyze data with an abnormal distribution. Continuous data are presented as median and range, and the Mann–Whitney *U*-test was utilized to assess the significance of differences for continuous data. A *p*-value of less than 0.05 was considered statistically significant.

## Results

### Demographic

Table 1 illustrates the demographic data of 11 female patients, aged between 36 and 54 years (median age 45 years). All patients were premenopausal with a history of fertility. Among them, 8 patients (72.7%) had a previous history of uterine fibroids, and 10 patients (90.9%) had a history of gynecological or obstetric surgery. None of the patients had undergone other surgeries in the past. Among the 5 patients who had undergone myomectomy, the median time since the previous myomectomy was 60 months (Table 1). The presenting symptoms were non-specific, with abnormal uterine bleeding (AUB) present in 6 cases, and two patients experiencing associated cardiovascular symptoms (Table 1).

**Table 1:** Demographic data of the patients

History and symptoms	Description	Numbers
Main symptoms	Abnormal uterine bleeding	6
	None	3
	Panic and chest tightness combined with syncope	1
	Abdominal distension, chest tightness and edema of lower limbs	1
History of uterine leiomyoma	Yes	8
	No	3
Past surgery	Cesarean section (CS)	4
	Cesarean section plus myomectomy	4
	Myomectomy	1
	CS plus excision of benign ovarian cyst and bilateral tubal excision	1
	None	1
Interval between myomectomy	Median (range) months	60 (10–120)
	No myomectomy	6
CA125 (U/ml)	Median (range)	20.8 (12.6–89.5)
UL size (mm)	Median (range)	57.5 (50–100)
Site of UL	LSU or cervix	6
	Uterus body	3
	Uterus corner	1
	Cervix	1
Preoperative diagnosis of IVL	Yes	7
	No	4

### Preoperative assessment

**Fig. 1:**
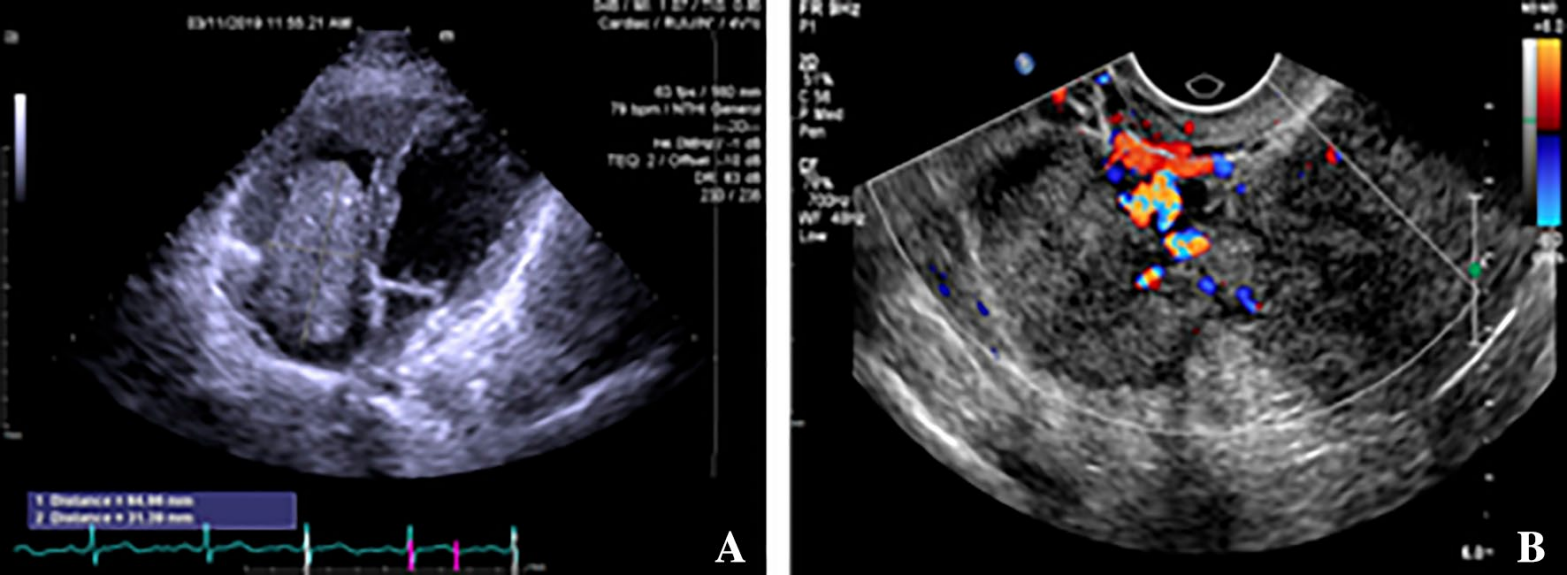
Preoperative ultrasound of Case 8. **A** Abnormal echo mass in the right heart cavity. **B** Tortuous paracervix vessels

Tumor indicator CA-125 showed a slight elevation (Table 1), while the other indicators were normal. Two patients were initially seen by a cardiologist, and echocardiography revealed an abnormal floating mass in the right atrium. During diastole, part of the mass entered the right ventricle, possibly originating from the inferior vena cava (Fig. 1A). All patients underwent pelvic ultrasound, except for Case 10. Among the diagnosed cases (90.9%), multiple uterine fibroids were observed, with a maximum diameter of 50 mm and above. Most uterine fibroids were found in the lower segment of the uterus or the cervix in 7 patients (Table 1). Blood flow signals were detected around the mass in all ultrasound examinations. Additionally, pelvic ultrasound reported tortuous and thickened parauterine/uterine veins with a diameter of 10–35 mm in 4 patients (Fig. 1B) (Table 2). One patient without uterine fibroids underwent diagnostic curettage due to AUB, which resulted in a pathological diagnosis of endometrial atypical hyperplasia.

**Table 2:** Preoperative USD/CT/MRI

Case	USD (diameter of uterine vein)	Lesions in MRI	Lesions in CT
1	Normal	Normal	Normal
2	Normal	Undone	Undone
3	Normal	Right UV and IV	RIV
4	Normal	Undone	Undone
5	Normal	Uterine fibroids	Normal
6	Normal	Undone	Undone
7	Normal	Both sides of UV and IV	Both sides of IV
8	16 mm	Left UV and IV	Left IV-IVC-right atrium
9	35 mm	Both sides of UV and IV	Both sides of IV-IVC-RA
10	28 mm	Right UV and IV	Right IV-IVC
11	10 mm	Both sides of UV	Normal

*UV* Uterine vein; *IV* iliac veins; *IVC* inferior vena cava

**Fig. 2:**
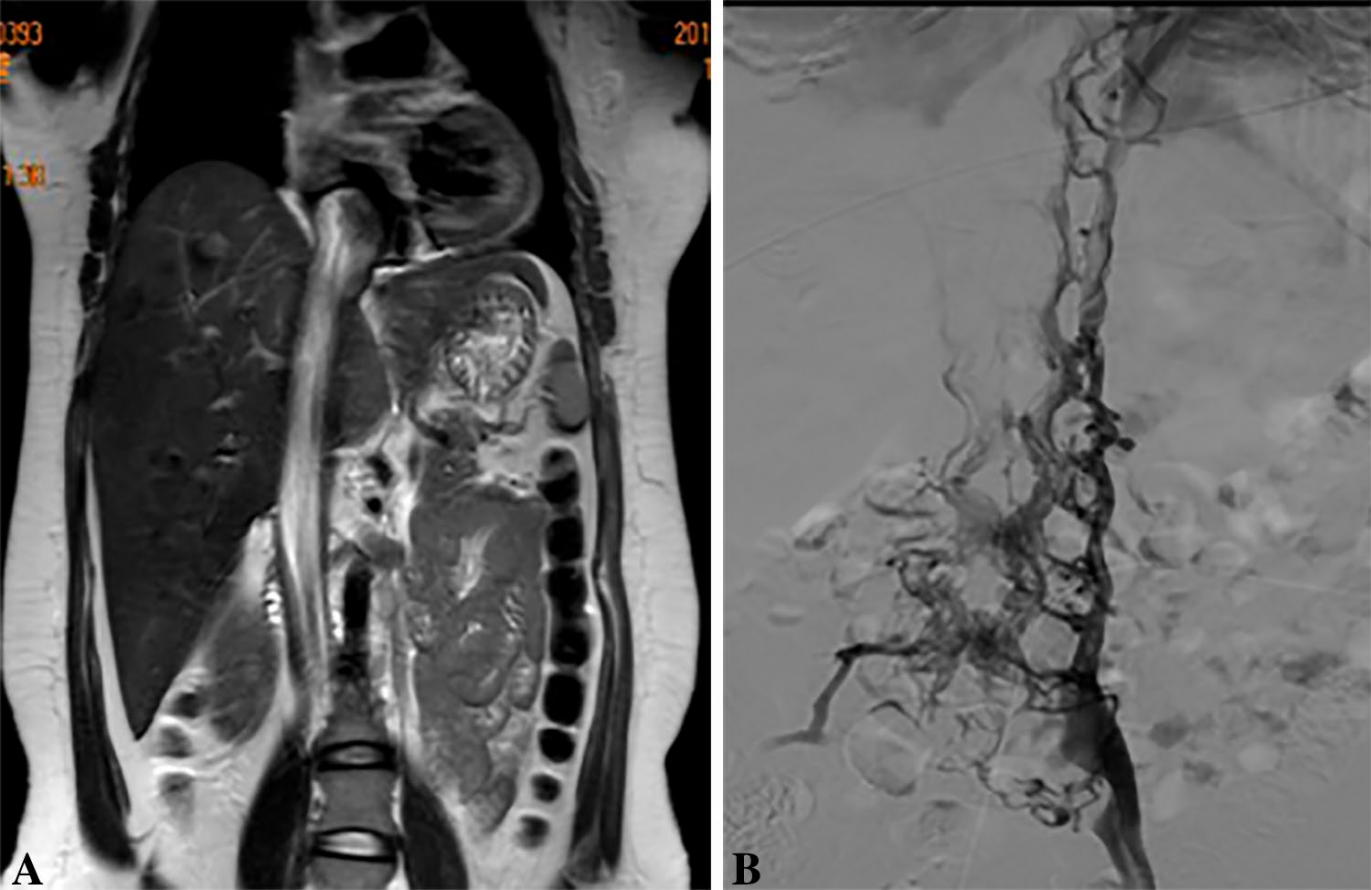
MR and inferior vena cava angiography of case 8. **A** Occupied space in the right atrium and involved inferior vena cava. **B** Inferior vena cava was not clearly shown, and the blood flowed back via the vertebral vein and azygos vein to the atrium

Enhanced magnetic resonance (MR) or computed tomography (CT) evaluations of the chest, abdomen, and pelvis were performed in 8 patients, because4 of them were suspected of having IVL (as indicated by pelvic ultrasound showing dilation and distortion of the parauterine/uterine vein), 3 were suspected of uterine sarcoma (due to uterine mass combined with elevated CA125), and 1 was suspected of having endometrial atypical hyperplasia. Among these 8 patients, MR revealed lesions in the parauterine/uterine vein or iliac veins in 6 patients (75.0%) (Table 2). Five patients (62.5%) had lesions outside the uterus in CT: 2 patients had lesions limited to the internal iliac vein, 1 patient had lesions extending from the right iliac vein to the inferior vena cava, and 2 patients had lesions extending from the inferior iliac vein to the right atrium (Table 2). MR imaging showed one patient with a completely blocked inferior vena cava (Fig. 2A), and angiography confirmed that the main trunk of the inferior vena cava was completely obstructed, with blood flow returning to the atrium via the vertebral vein and azygos vein (Fig. 2B).

### Surgical treatment

#### Stage I

According to the classification method of Ma et al. [3], there were 8 patients (8/11, 72.73%) at stage I. Three patients were diagnosed with IVL before surgery. Among them, 1 patient with lesions in the parauterine/uterine vein underwent a hysterectomy plus bilateral salpingo-oophorectomy, and resection of the lesion in the parauterine/uterine vein. Two of the 3 patients with lesions extending from the parauterine/uterine vein to the internal iliac vein also underwent hysterectomy and resection of the lesion in the parauterine/uterine and internal iliac vein, while 1 patient chose to undergo only bilateral salpingo-oophorectomy, as she wanted to keep both ovaries (Table 3). None of these patients required ICU admission after surgery.

**Table 3:** Surgical details

Variables	Description	Quantity
Blood loss (ml)	Median (range)	150 (50–4000)
Blood transfusion (ml)	Median (range)	500 (200–2400)
	None	5
ICU stay (days)	Median (range)	2 (2–5)
	None	8
Postoperative stay (days)	Median (range)	5 (3–15)
Classification	I	8
	II	1
	III	2
Surgery	Laparoscopic PH plus BS	4
	PH plus BS and resections of lesion in right internal iliac vein	2
	PH plus BS and resections of lesions in IVC under CPB	2
	Myomectomy	1
	PH plus BS	1
	PH plus BS and resections of lesions in IVC	1

*PH* Panhysterectomy; *BS* bilateral salpingo-ophorectomy; *IVC* inferior vena cava; *CPB* cardio-pulmonary bypass

Five patients at stage I were not diagnosed with IVL before surgery, and 4 of them underwent laparoscopic total hysterectomy plus bilateral salpingectomy, while 1 patient underwent transabdominal myomectomy. No para-uterine endovascular lesions were observed visually during surgery, but IVL was confirmed by postoperative pathology.

#### Stage II and III

**Fig. 3:**
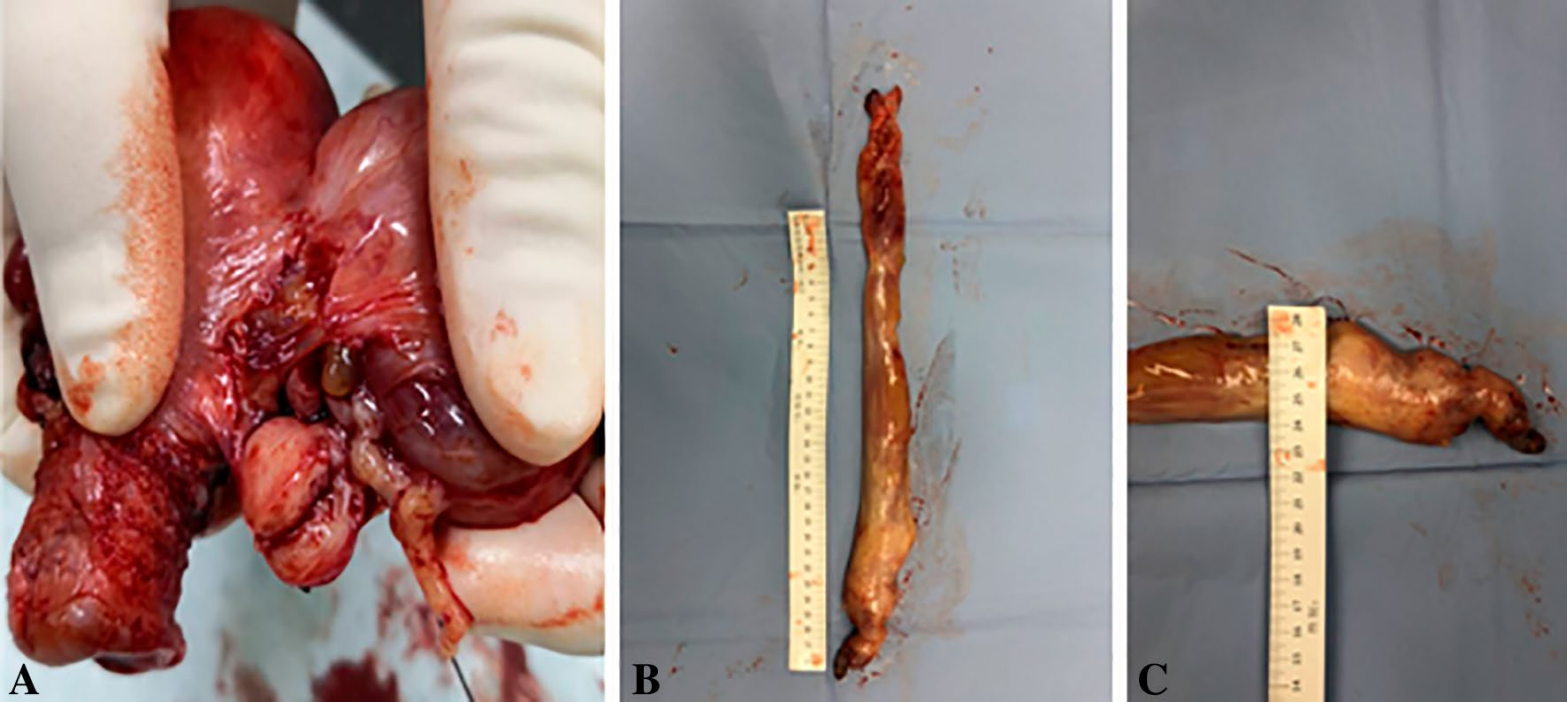
Gross view of postoperative specimen of Case 8. **A** Intravascular tumors. **B** and **C** Tumors through Inferior vena cava—right atrium

Among the 11 patients, there was 1 at stage II (1/11, 9.09%) and 2 at stage III (2/11, 18.18%). In stage II, the lesion extended from the parauterine/uterine vein to the internal iliac vein, common iliac vein, and reached the inferior vena cava. In stage III, the lesion extended from the parauterine/uterine vein to the internal iliac vein, common iliac vein, inferior vena cava, and reached the right atrium. These cases were thoroughly discussed and evaluated preoperatively by multidisciplinary teams (Departments of gynecology, radiology, anesthesiology, cardiology, and vascular surgery). One-stage simultaneous surgery was planned for all three patients, including resections of tumors inside the circular system, hysterectomy, and bilateral salpingo-oophorectomy. Two patients with lesions in the right atrium underwent combined thoracic and abdominal incision surgery. Due to the width of the tumor in the right atrium being greater than the diameter of the inferior vena cava, the lesion from the inferior vena cava to the right atrium was removed from the right atrium under cardiopulmonary bypass (Fig. 3). All 3 patients received blood transfusions and were admitted to the ICU after surgery (Table 3).

Of the 11 patients, 6 (54.55%) had lesions limited to uterine/parauterine veins, while the other 5 (45.45%) had lesions invading the internal iliac vein and above. Patients with lesions invading the internal iliac vein and above experienced significantly increased intraoperative blood loss and postoperative hospital stay compared to those with lesions limited to parauterine/uterine veins (median blood loss 50 ml vs. 1300 ml, *p* = 0.005; median hospital stay 10 vs. 4 days, *p* = 0.026).

### Follow-up

All 11 patients in the study were confirmed to have IVL (intravenous leiomyomatosis) pathologically after undergoing surgery. Interestingly, 5 of these patients had not been diagnosed with IVL before the operation. However, the presence of IVL in the blood vessels of the uterine body was confirmed through pathological analysis of tissue sections. Furthermore, immunohistochemistry revealed that all patients’ IVL lesions tested positive for estrogen receptor (ER) and progesterone receptor (PR).

Additionally, all patients were pathologically confirmed to have uterine leiomyoma, and other pathological diagnoses included endometrial atypical hyperplasia, adenomyosis, and endometriosis of the ovary (Table 4). A month after the surgery, all patients underwent reexamination, and it was found that two patients at stage II–III still had residual lesions in the internal iliac vein.

**Table 4:** Pathology and follow-up

Postoperative follow-up	Description	Numbers
Pathology other than uterine leiomyoma	None	6
	Adenomyosis	3
	Endometriosis of ovary	1
	Endometrial atypical hyperplasia	1
Postoperative treatment	None	7
	GnRH injection	3
	L-Oophorectomy	1
Follow-up time	Months	46 (18–59)
Disease progression	No	9
	Yes (21 months)	1
	Yes (36 months)	1

Upon diagnosing IVL, one patient with atypical endometrial hyperplasia underwent a second operation of laparoscopic oophorectomy. In total, three patients received hormone therapy with gonadotropin-releasing hormone agonists (GnRHa) for six months to prevent disease progression. This included one patient who retained the uterus and two patients who had residual disease in the internal iliac vein after the initial surgery.

The patients were regularly followed up at intervals ranging from 3 to 12 months, with a median follow-up period of 46 months (as of February 2023). Among the nine patients with no residual lesions, there was no evidence of recurrence. However, the two patients with residual lesions in the internal iliac vein showed disease progression. One of these cases (Case 7) experienced disease progression at 21 months after surgery, while the other case (Case 9) showed disease progression at 36 months after surgery. CT scans revealed that the lesion extended from the internal iliac vein to the common iliac vein and the inferior vena cava but did not reach the heart. It is worth noting that both patients remained asymptomatic despite disease progression.

In response to the disease progression, one patient (Case 7) underwent a resection of the intravenous tumor due to the rapid progression of the disease. Conversely, the other patient (Case 9) was treated with letrozole without undergoing surgery. At the last follow-up in February 2023, which occurred 20 months after the second surgery for Case 7 and 4 months after taking letrozole for Case 9, the lesions of both patients remained stable.

## Discussion

Intravenous leiomyomatosis (IVL) is a rare benign disease that presents as a biologically aggressive tumor [1]. Two hypotheses have been proposed in the literature regarding its etiology: it originates from neoplastic smooth muscle cells of the uterus invading the veins of the reproductive system [10] or the proliferation of smooth muscle cells in the tunica media of blood vessels [11]. Among the 11 patients reported in this paper, 8 (72.7%) had a history of uterine fibroids, and 5 (45.5%) had undergone myomectomy. Post-surgery, all patients were pathologically confirmed to have uterine fibroids. Furthermore, in 5 patients with lesions invading the internal iliac vein and beyond, the extension pattern was similar to the return path of parauterine/uterine veins, where the lesion extended from the uterine vein to the internal iliac vein, common iliac vein, inferior vena cava, and even reaching the right atrium. Based on the evidence discussed, it is considered that IVL originates from neoplastic smooth muscle cells of the uterus invading the veins of the reproductive system. The literature suggests that HMGA2 protein expression along with the translocation of t12;14 q15;q24 is more common in patients with IVL [12], which may play an important role in allowing the invasion of leiomyomas into the vascular system.

IVL typically presents with nonspecific symptoms, and its diagnosis heavily relies on imaging, such as pelvic ultrasound, enhanced CT, and MRI [6, 13]. Pelvic ultrasound is the most convenient, minimally invasive, and widely used examination method for diagnosing gynecological diseases. In our experience, IVL lesions usually extend upward via the uterine vein, and tortuous and thickened parauterine/uterine veins can be observed under pelvic ultrasound in most patients. However, it’s worth noting that pelvic ultrasound can only assess the pelvic veins, and its accuracy often depends on the experience of the sonographer. Enhanced CT or MRI is more helpful in evaluating the internal iliac-common iliac-inferior vena cava venous system, assessing the extent of the lesion, and determining operative procedures [14, 15]. Clinicians can interpret these images themselves, eliminating the need to rely solely on the sonographer’s diagnosis. Due to resource limitations, CT/MR is not suitable for routine screening, and thus, pelvic ultrasound can be used for the initial screening of IVL. If pelvic ultrasound reveals uterine fibroids combined with tortuous and thickened parauterine/uterine veins in a patient, the possibility of IVL should be highly suspected, and further confirmation through enhanced CT or MR is necessary. Additionally, echocardiogram evaluation is often used to assess heart cavity lesions, and the mass in IVL is highly mobile and can be seen originating from the inferior vena cava [2, 16].

The best treatment of IVL is complete tumor resection, combined with a hysterectomy and Bilateral salpingo-ophorectomy [4, 7–9], whether the lesion is completely resected is the most important factor for recurrence [17, 18]. For patients with lesions above the inferior vena cava, multidisciplinary treatment is needed, especially the combined operation of general surgeons and cardiac surgeons during the operation. For patients with lesions entering the cardiac cavity, it is usually necessary to evaluate the maximum diameter of the cardiac tumor and the diameter of the entrance of the inferior vena cava [14, 15]. IVL lesions are usually relatively strong and can withstand high tensile strength without breaking [19]. If the maximum diameter of the cardiac tumor is less than the diameter of the inferior vena cava entrance, the tumor can be pulled out after the inferior vena cava is cut through a single abdominal incision. If the maximum diameter of the cardiac tumor is larger than the diameter of the inferior vena cava, combined thoracoabdominal surgery should be performed under cardiopulmonary bypass to open the heart and remove the lesion, and chest and abdominal surgery can usually be completed in one session. [14, 15, 20, 21]. Out of the 11 patients, 6 (54.55%) had lesions limited to the parauterine/uterine area and were operated on via transabdominal or laparoscopic surgery, resulting in less intraoperative bleeding. However, 2 patients had lesions above the internal iliac vein, but not reaching the inferior vena cava, which significantly increased blood loss, necessitating blood transfusions. Nonetheless, these cases did not generally require ICU admission after surgery. Patients with lesions invading the internal iliac vessels or extending above experienced significantly higher perioperative blood loss, often requiring a large amount of blood preparation and ICU admission for further treatment after surgery. Two patients had residual lesions after surgery (including 1 patient with stage I lesion invading the internal iliac vein and 1 patient with stage III). Both patients showed disease progression during follow-up. Therefore, patients without lesions in the internal iliac vein and above have lower surgical risk, higher surgical resection rates, and a better prognosis.

IVL mostly occurs in premenopausal women [22], In this study, all 11 patients were premenopausal. It is generally considered an estrogen-dependent tumor due to the positive expression of estrogen and progesterone receptors in most cases, as revealed by immunohistochemistry [23]. Currently, the main treatment methods for IVL are surgery and anti-estrogen therapy [4, 7, 8, 14]. Anti-estrogen therapies reported in the literature include sBSO-based(Bilateral salpingo-oophorectomy) surgery [7] and Anti-estrogen hormonedrugs [24–26] (GnRH, Aromatase inhibitors, etc.), These treatments may involve preoperative adjuvant therapy to reduce the lesion size and facilitate surgery [7], as well as postoperative adjuvant therapy to lower the recurrence rate of the disease [24–26]. However, complete resection of the lesion remains crucial in preventing recurrence [22]. In this study, Case 10 underwent GnRHa therapy for six months before surgery, but no significant reduction in the lesion was observed during follow-up. Two patients with progressive disease (Case 7, Case 9) had residual disease one month after surgery, despite receiving GnRHa therapy for 6 months, indicating disease progression. One patient with stable disease (Case 9) experienced improvement after switching to letrozole, while another patient (Case 7) faced rapid disease progression due to preserved ovaries during surgery, necessitating bilateral resection of ovarian and venous lesions after recurrence. In contrast, Case 6 was not diagnosed with IVL before surgery, and an open myomectomy was performed for uterine fibroids. The lesion was completely removed, and no IVL lesions were found during intraoperative exploration. The patient received GnRHa therapy for 6 months, and no signs of disease recurrence were found during postoperative follow-up, eliminating the need for a second operation. Based on these experiences, complete resection of the lesion appears to be the most effective way to prevent IVL recurrence and progression.

**Fig. 4:**
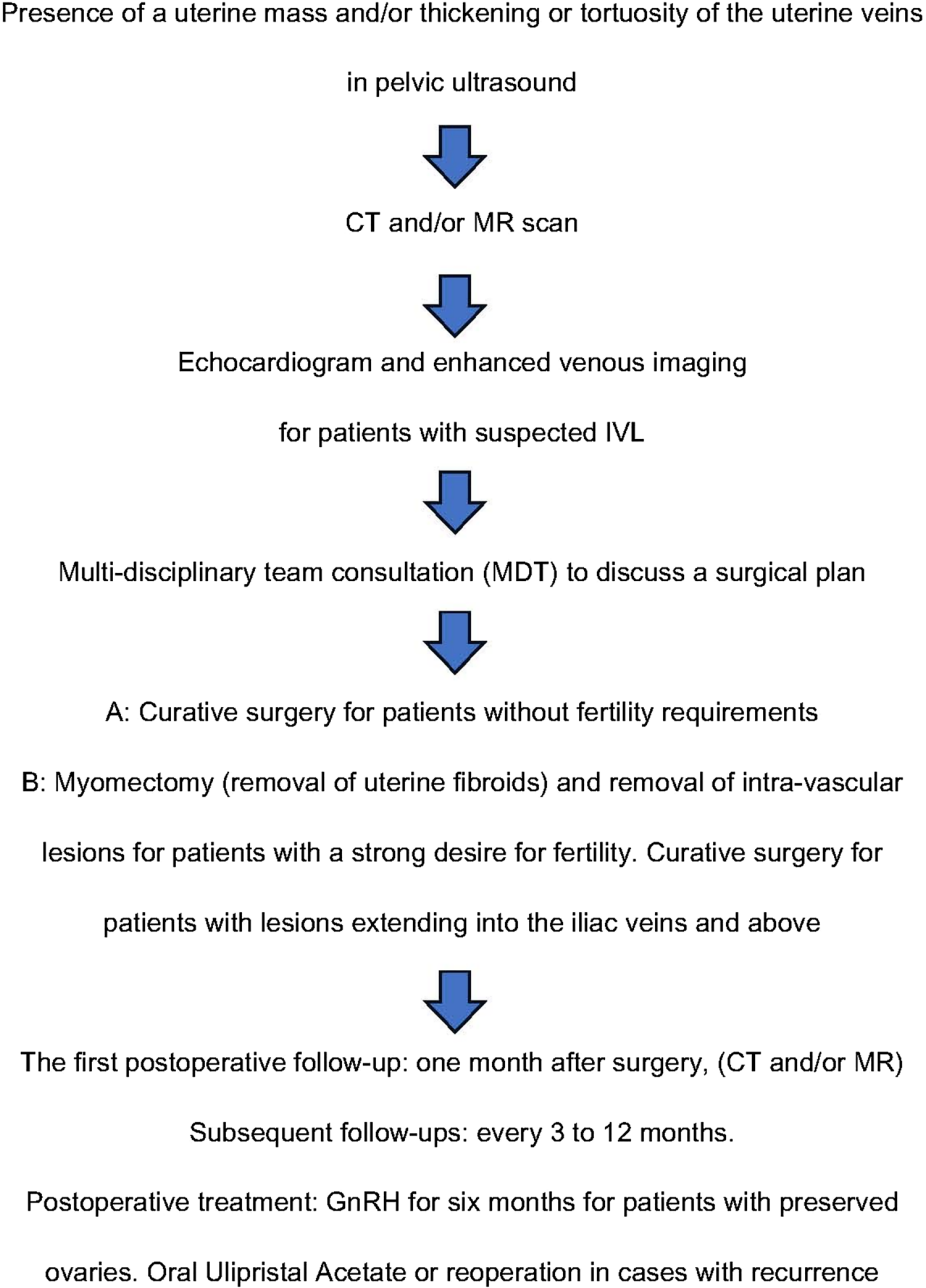
Flowchart for overall management of IVL

This article summarizes our diagnostic and treatment experiences with IVL, covering the onset symptoms and signs, diagnosis, treatment options, and follow-up recurrence. It has enriched the clinical understanding of this disease among medical professionals and offers valuable insights for the diagnosis and treatment of the rare disease IVL. To enhance clarity, we have created a flowchart to facilitate better understanding (Fig. 4).

The strengths of this article lie in its systematic summary of the rare disease IVL, encompassing everything from initial symptoms and signs to diagnosis, treatment options, and follow-up recurrence. There are currently few articles that provide such a comprehensive overview of this disease. This article summarizes the diagnostic and treatment experiences of 11 patients, which is a notable advantage for a rare disease. It’s important to acknowledge the limitations of this study. As IVL is a rare disease, our analysis is retrospective and based on a small number of cases. Additionally, most of the cases collected were at stage I, with no summary cases of stage IV. Further large-sample data or prospective studies are needed to confirm our conclusions.

In conclusion, intravenous leiomyomatosis (IVL) is most commonly found in premenopausal women with uterine masses. The majority of IVL lesions are confined to the pelvic cavity (Stage I). Pelvic ultrasound is useful for early screening, while enhanced CT or MRI aids in diagnosing and evaluating the extent of venous lesions. Complete resection of the lesion is imperative to prevent IVL recurrence. Patients with lesions invading the internal iliac vein and above experience increased intraoperative blood loss and postoperative hospital stays compared to patients with lesions limited to the parauterine/uterine veins. Cases involving lesions in or above the inferior vena cava require a multidisciplinary team approach and may benefit from simultaneous surgery.

## Data Availability

The datasets used and/or analyzed in the study can be accessed by the corresponding author at reasonable request.
